# Human H5N2 Avian Influenza Infection in Japan and the Factors Associated with High H5N2-Neutralizing Antibody Titer

**DOI:** 10.2188/jea.JE2007446

**Published:** 2008-08-07

**Authors:** Tsuyoshi Ogata, Yoshinao Yamazaki, Nobuhiko Okabe, Yosikazu Nakamura, Masato Tashiro, Noriko Nagata, Shigeyuki Itamura, Yoshinori Yasui, Kazutoshi Nakashima, Mikio Doi, Youko Izumi, Takashi Fujieda, Shin’ichi Yamato, Yuichi Kawada

**Affiliations:** 1 Chikusei Health Center, Ibaraki Prefectural Government.; 2 Ibaraki Prefectural Institute of Public Health.; 3 Infectious Disease Surveillance Center, National Institute of Infectious Diseases.; 4 Department of Public Health, Jichi Medical University.; 5 Department of Virology, National Institute of Infectious Diseases.; 6 Department of Health and Welfare, Ibaraki Prefectural Government.

**Keywords:** Influenza, Human Influenza, H5N2, Neutralization Tests, Vaccination

## Abstract

**Background:**

H5N2 avian influenza virus infection of humans has not been reported thus far. The first H5N2 avian influenza infection of poultry in Japan occurred in Ibaraki.

**Methods:**

The subjects were workers at 35 chicken farms in Ibaraki Prefecture, where the H5N2 virus or antibody was isolated from chickens. None of the subjects exhibited influenza symptoms. The H5N2-neutralizing antibody titers of the first and second paired sera samples were compared. To investigate the possible factors for this increase, the H5N2-neutralizing antibody titer (1:40 or more) was calculated for the second samples. A logistic regression analysis was performed to examine the association of these factors with H5N2-neutralizing antibody positivity.

**Results:**

We performed Wilcoxon matched-pairs signed-ranked test on data collected from 257 subjects, and determined that the H5N2 antibody titers of the second paired sera samples were significantly higher than those of the first samples (*P* < 0.001). The H5N2 antibody titers of paired sera of 13 subjects without a history of seasonal influenza vaccination within the previous 12 months increased 4-fold or more. The percentage of antibody positivity was 32% for subjects with a history of seasonal influenza vaccination (28% of all subjects) and 13% for those without a history of the same. The adjusted odds ratio of H5N2-neutralizing antibody positivity was 4.6 (95% confidence interval: 1.6-13.7) for those aged over 40 and 3.1 (95% confidence interval: 1.6-6.1) for those with a history of seasonal influenza vaccination within the previous 12 months.

**Conclusion:**

The results suggest that this may have been the first avian influenza H5N2 infection of poultry to affect humans. A history of seasonal influenza vaccination might be associated with H5N2-neutralizing antibody positivity.

## INTRODUCTION

Highly pathogenic avian influenza is currently prevalent in poultry worldwide. The H5N2 as well as H5N1 subtype of the avian influenza virus is also found to infect poultry. Highly pathogenic avian influenza infection may become pandemic through a virus mutation or reassortment in the future; therefore, control of avian influenza infection has been recognized as a public health issue of importance on a global scale.

Ibaraki Prefecture is located in the northeast of Tokyo, Japan, and has a population of 3 million. The chicken population in chicken farms in this prefecture is 11 million-the largest among all the prefectures in Japan. In June 2005, the anti-H5N2 avian influenza A antibody was isolated from a chicken on a farm in this prefecture for the first time in Japan.^1^^-^^3^ By December 2005, the virus was isolated or an anti-H5 antibody was identified from chickens in 40 chicken farms in Ibaraki Prefecture and in 1 chicken farm in Saitama Prefecture. The isolates were closely related to the Guatemala strain^2^^-^^4^ with high homology with regard to the nucleotide sequences, and the prototype strain was designated A/chicken/Ibaraki/1/2005(H5N2).^5^ As in earlier cases in Taiwan and South Korea, in Japan too, the chickens at these farms where the H5N2 virus or antibody was detected were destroyed and disposed of. In Ibaraki, 5.7 million chickens were culled.

To date, no cases of H5N2 avian influenza type A virus infection in humans have been reported.^6^ Considering the fact that the avian influenza H5N1 virus has been transmitted from poultry to humans,^7^ it is not surprising that H5N2 avian influenza virus infections would also be transmitted from infected poultry to human beings.

In the case of inhabitants with high avian influenza antibody titers, the cross-reactivity by infection or inoculation with a seasonal influenza virus as well as an avian influenza infection is considered to be a possible cause of the increased antibody titer. Myers et al, however, reported that human influenza vaccination in the previous 3 years was not associated with elevated titers against viral subtypes H5, H6, H7, and H9.^8^ No studies have reported an increase in the H5N2 avian influenza antibody titers as a result of seasonal influenza vaccination of humans.

This investigation was undertaken to ascertain whether there were suspected cases of H5N2 human infection in H5N2 virus-positive chicken farms. In addition, we examined whether the increased antibody titer was associated with factors such as a history of seasonal influenza vaccination.

## METHODS

### Subjects

In Ibaraki Prefecture, from June through December 2005, the virus was isolated or anti-H5 antibody was identified from chickens on 40 chicken farms. No chickens in these yards exhibited symptoms, except for a temporary drop in the egg-laying rate at the farm where the antibody was first detected. A total of 332 workers (210 males and 122 females) were employed on these 40 farms, and they were at risk of exposure to the infected chickens.

The subjects were 311 workers (199 males and 112 females) who worked at 35 chicken farms where the virus was isolated or anti-H5 antibody was identified in chickens from June through November 2005; they were at risk of exposure to the virus. No subjects had symptoms that indicated an influenza viral infection. Human influenza rapid diagnostic tests and human influenza H5 virus-specific PCR showed that all the pharyngeal swabs of workers on the chicken farms were negative for the virus.^1^^,^^2^ In the 35 farms, the number of exposed farm workers was over 20 in 4 chicken farms.

### Data Collection and Serological Tests

When the H5N2 virus or antibody was detected in chickens on a farm, public health nurses affiliated with any of the 4 public health centers of the Ibaraki Prefectural Government immediately checked on the people associated with the farm, using a questionnaire to record age, sex, current symptoms, history of vaccination against seasonal influenza since 2004, and the time in case of vaccination.

During the 2004-2005 influenza season, the composition of trivalent vaccines with which most of the chicken farm workers were vaccinated was A//New Caledonia/20/99(H1N1), A/Wyoming/3/2003(H3N2), and B/Shanghai/361/2002, while the composition for the 2005-2006 influenza season during which some of the chicken farm workers were vaccinated was A//New Caledonia/20/99(H1N1), A/New York/55/2004(H3N2), and B/Shanghai/361/2002.

Paired serum samples were collected from the subjects by public health nurses employed by the government at intervals of at least 4 weeks and up to 2 months. The peak of the influenza antibody titers generally occurs 4-7 weeks after an infection.^9^ The first blood samples of paired sera were obtained immediately after the H5N2 virus or antibody was detected in chickens on the aforementioned chicken farm. In most cases, however, these chickens exhibited no symptoms and the first samples of paired sera were collected when the H5N2 virus was no longer detected in them. A certain period of time might have elapsed after exposure to the infected chickens.

Measurement of the neutralization titer of the antibody against avian influenza virus A/chicken/Ibaraki/1/2005(H5N2) was performed at the National Institute of Infectious Diseases according to the method previously described.^10^ The neutralization titer was expressed as the highest dilution rate that inhibited a 50% tissue culture infection dose. The antibody titers were measured at least twice for confirmation.

### Statistical Analyses

The proportions of subjects with seasonal influenza vaccination were calculated according to the subject’s age. Chi-square tests were used to examine the differences with regard to sex and histories of seasonal influenza vaccination within the previous 12 months, among the age classes. The number of chicken farms where the H5N2 virus or antibody was detected in the chickens and the number of employees on these farms were tabulated for each month.

H5N2 antibody titers of the first and second paired sera samples were compared for each subject and totaled. To determine whether a subject was infected by the H5N2 virus, the following criterion was considered noteworthy: a 4-fold or greater rise in the neutralizing antibody titer in paired sera. The geometric mean of the antibody titer of the first and second samples of the subjects’ paired sera was calculated and then compared using the Wilcoxon matched-pairs signed-rank test.

Positive rates of the H5N2-neutralizing antibody in the second paired sera samples were also calculated according to age and history of seasonal influenza vaccination within the previous 12 months. A neutralizing antibody titer of 1:40 or more was used as a criterion for determining the positivity of a neutralizing antibody because the criterion for determining the positivity of an H5N2-neutralizing antibody for a single serum had not yet been established.

Crude odds ratios (ORs) and their 95% confidence intervals (CIs) were estimated by variables. Unconditional logistic regression analyses were performed using sex, categorized age, chicken farm categorized by the number of workers, and history of seasonal influenza vaccination as independent variables, and adjusted ORs and their 95% CIs were estimated by variables, to examine their association with a neutralizing antibody that was positive against the H5N2 virus.

Statistical analyses were performed using Dr. SPSS^®^ 11.0J for Windows software (SPSS Inc., Chicago, IL, USA).

### Ethical Issues

The Ibaraki Prefectural Government established a research committee for the implementation of this study. Approval of the Ibaraki Joint Committee for Ethical Review of Epidemiological Research was obtained on April 17, 2006. A written explanation was provided to each subject and his signed consent was obtained.

## RESULTS

We excluded subjects who failed to provide paired sera or appropriate data. Thus, 257 people (83%) were analyzed; Table 1 shows their characteristics. Seasonal influenza vaccinations had been administered to 28% of the subjects. No significant differences were found with regard to the history of seasonal influenza vaccination among different age classes. A smaller number of females were included in the younger age groups.

**Table 1.** Charasteristics of the study subjects

	Age (year)							Subjects with a history of seasonal influenza vaccination within the previous 12 months (%)

	<29	30-39	40-49	50-59	60<	Total	*P*-value*	
Total	32	33	39	92	61	257		71 (28)

Sex								
Males	26	27	28	56	34	171	0.02	44 (26)
Females	6	6	11	36	27	86		27 (31)

History of seasonal influenza vaccination in the previous 12 months (%)								
Yes	9 (28)	6 (18)	10 (26)	27 (29)	19 (31)	71 (28)	0.72	
No	23 (72)	27 (82)	29 (74)	65 (71)	42 (69)	186 (72)		

* : *P*-value for the difference among age classes according to variables by the Chi-square test

Table 2 shows the number of chicken farms where the H5N2 virus or antibody was detected in the chickens for each month and the number of persons employed at these farms.

**Table 2.** Number of chicken yards where the H5N2 virus or antibody was detected in the chickens and the number of subjects employed on these yards according to the month, in 2005.

	June	July	August	September	October	November	Total
Chicken yards*	6	3	12	9	1	4	35
Subjects	25	18	120	37	5	52	257

* : No. of chicken yards where the H5N2 virus or antibody was detected in the chickens for a given month

The interval between the time at which the H5N2 virus or antibody was detected in the chickens on these farms and the time at which the first sample was collected from the workers was an average of 1.6 days and a maximum of 5 days. The shortest and longest intervals for sampling of paired sera were 28 and 63 days, respectively. Table 3 shows the relationship between the first and second samples of the paired sera in terms of the H5N2 antibody titers. A 4-fold or greater increase in antibody titers was observed in the paired sera of 20 chicken farm employees; 13 (65%) had no history of seasonal influenza vaccination in the previous 12 months. In 65 subjects (including the abovementioned 20), either a 4-fold or greater increase in the antibody titer of paired sera was observed, or the antibody titer of the first or second samples of paired sera was 1:40 or greater. H5N2 antibody titers of the second samples of paired sera of the subjects were significantly higher than those of the first samples when examined by a Wilcoxon matched-pairs signed-rank test (*P* < 0.001).

**Table 3.** Relationship between the first and second paired sera samples of subjects, with regard to the H5N2 antibody titers.

Neutralizing antibody									Four-fold or greater increase in antibody titers	

First samples		Second samples								
	
		<10	10	20	40	80	160	320<	Total	Without a history of seasonal influenza vaccination within the previous 12 months
<10	80	44	30	5	1				6	5
10	111	14	50	36	10	1			11	8
20	29		6	12	10	1			1	0
40	21		4	6	6	4	1		1	0
80	10	1	1		3	2	2	1	1	0
160	3					2	1		0	0
320<	2					1		2	0	0

Total	257	59	91	59	30	11	4	3	20	13

Geometric mean titer	11.4				14.0				*P*-value* = 0.000	

* : *P*-value for the difference between the first and second samples based on Wilcoxon matched-pairs signed-rank test

Table 4 shows the percentage of positive H5N2-neutralizing antibody titers of the second samples stratified by age and history of seasonal influenza vaccination within the previous 12 months. A positive rate of 32% was observed for subjects with a history of seasonal influenza vaccination within the previous 12 months, and it was 13% for those without a history of the same. In each of the age classes, the positive rate was also higher for those with a history of seasonal influenza vaccination within the previous 12 months than for those without. The positive rate was more likely to be higher among subjects aged over 40 than among those under 40. Therefore, the subjects were divided into those who were older than 40 and under 40 years of age. There were no antibody-positive subjects among those who were under 40 and did not have a history of seasonal influenza vaccination within the previous 12 months. Multivariate logistic regression analyses revealed that the adjusted OR for the positivity of H5N2-neutralizing antibody was 4.6 (95% CI: 1.6-13.7) for those aged over 40 years and 3.1 (95% CI: 1.6-6.1) for those with a history of seasonal influenza vaccinations within the previous 12 months (Table 5).

**Table 4.** Positive rate of H5N2-neutralizing antibody titer of the second paired sera samples according to age and histories of seasonal influenza vaccination within the previous 12 months.

	Neutralizing antibody positive* / Total (%)					

Age (year)	<29	30-39	40-49	50-59	60<	Total
Total	4/32 (13)	0/33 (0)	10/39 (26)	23/92 (25)	11/92 (18)	48/257 (19)

History of seasonal influenza vaccination within the previous 12 months						
Yes	4/9 (44)	0/6 (0)	4/10 (40)	10/27 (37)	5/19 (26)	23/71 (32)
No	0/23 (0)	0/27 (0)	6/29 (21)	13/65 (20)	6/42 (14)	25/186 (13)

* : Subjects whose neutralizing antibody titer for the second paired sera samples is 1:40 or more

**Table 5.** Odds ratios for positivity of H5N2-neutralizing antibody titers according to variables.

	No. at risk	No. of cases positive for neutralizing antibody* (%)	Crude odds ratio (95% CI)	Adjusted odds ratio^†^ (95% CI)
Sex				
Male	171	32 (19)	1.00 (reference)	1.00 (reference)
Female	86	16 (19)	0.99 (0.51-1.93)	0.73 (0.35-1.51)

Age (year)				
<39	65	4 (6)	1.00 (reference)	1.00 (reference)
40<	192	44 (23)	4.5 (1.56-13.2)	4.6 (1.56-13.7)

Number of workers in the chicken farm				
<19	157	30 (19)	1.00 (reference)	1.00 (reference)
20<	100	18 (18)	0.92 (0.49-1.78)	0.86 (0.42-1.76)

History of seasonal influenza vaccination within the previous 12 months				
Yes	71	23 (32)	3.1 (1.61-5.9)	3.1 (1.59-6.1)
No	186	25 (13)	1.00 (reference)	1.00 (reference)

* : Subjects whose neutralizing antibody titer for the second paired sera samples is 1:40 or more

† : Multivariable logistic regression model was adjusted for variables listed in the Table.

CI: confidence interval

## DISCUSSION

In this study, it has been shown that the H5N2 antibody titer increased significantly in the paired sera of workers at the chicken farms where the virus or specific antibody was identified, and that the H5N2 antibody titers of the paired sera of 13 workers at the chicken farms who had no history of seasonal influenza vaccination in the previous 12 months also showed a 4-fold or greater increase. Some subjects also had an H5N2 antibody titer of 1:40 or more. The results with a single serum sample showed that the positive rate for H5N2 neutralization antibody titer was significantly higher for those with a history of seasonal influenza vaccination in the previous 12 months and for those older than 40 years, although no significant differences were found with regard to the history of seasonal influenza vaccinations among age classes.

These data suggested that an avian-derived H5N2 influenza virus might infect humans through exposure to infected chickens. Four strains of avian influenza A virus-H5N1,^7^ H7N3,^11^ H7N7,^12^ and H9N2^13^-are known to infect humans. Although the H5N2 subtype of the avian influenza virus has been found in the United States, Mexico, Italy, Taiwan, and South Korea, thus far, there has been no report of this virus subtype infecting humans.^6^

The H5N1 virus has been responsible for the greatest number of human influenza cases with very severe disease and the highest mortality rate. Infection of humans with H7N3, H7N7, and H9N2 viruses has resulted in mild symptoms and, very rarely, in severe illness. The H5N2 influenza virus did not result in death of the infected chickens^1^^-^^3^, and no worker at the chicken farms in Ibaraki had symptoms that indicated a viral influenza infection. The increase in the antibody titers of the 13 workers was moderate: the amount of H5N2 virus on the chicken farms might be small or viral transmission or its pathogenic ability to inflict humans might be weak. On the other hand, the H5N2 virus in poultry later gained accentuated virulence in the United States^14^ and Mexico.^15^ Because this virus can become highly virulent against chickens, it may also prove to be highly virulent against humans. Although we should take utmost precautions against the H5N1 virus as the causative agent of an influenza pandemic, we cannot exclude the possibility that other influenza viruses may also cause the disease.

In the current study, the single serum specimen showed that the positive rate of the H5N2 neutralization antibody titer was significantly higher for the subjects with a history of seasonal influenza vaccination within the previous 12 months. This is the first observation of this phenomenon for the avian H5N2 virus. Immunity acquired via antibody targeting of the neuraminidase antigen is thought to be a possible cause of cross-reactivity of the human influenza virus antibody with that of an avian influenza virus.^16^ Gioia et al recently reported that seasonal vaccination could raise the neutralizing immunity against influenza (H5N1) in a large number of donors.^9^ A neutralizing antibody titer may only represent a nonspecific reaction, and it is possible to determine whether it is effective for the prevention of an avian influenza infection. Further examinations will be required to evaluate the significance of this finding.

The positive rate of the anti-H5N2-neutralizing antibody for subjects over 40 years of age was significantly higher when compared to that of subjects under 40 years. This could be because the aged may have been exposed to more diverse influenza viruses.^17^ The mortality rate of the younger generation was high for the Spanish flu (H1N1 subtype),^18^ which may have been due to their lack of exposure to the preceding pandemic virus. There were no neutralizing antibody-positive subjects among those aged under 40 and without a history of seasonal influenza vaccination within the previous 12 months. In any case, further study of the H5N2- and H5N1-neutralizing antibody titers in the serum is recommended; moreover, the impact of an influenza vaccination or one’s age on the antibody titer should be noted for animals and general inhabitants who have not been exposed to infected chickens.^19^

For determining H5N1 avian influenza infections, the World Health Organization (WHO) has adopted the following criterion: a person meeting the criterion for an H5N1-neutralizing antibody titer of 1:80 or more in a single serum specimen should be considered positive.^20^ However, if their age and seasonal influenza vaccination influence the antibody titer of avian influenza H5N2, they might also influence that of H5N1. Further examinations will be required to review the validity of this criterion by WHO.

This study had several limitations. First, data were extracted only from those chicken farms in Ibaraki Prefecture where chickens were infected with avian influenza; thus, the results might not be widely applicable to other areas or general inhabitants who are not exposed to infected chickens. Second, we were unaware of the time of infection of the chickens on each farm. In most cases, the chickens showed no symptoms and paired sera were collected when the H5N2 virus was no longer detected on the farm; therefore, a certain amount of time might have elapsed after exposure to the infected chickens and the infection of several subjects might not be detected through analysis of paired sera. Third, the study on the association between the variables and the neutralizing antibody titer for a single serum sample was based on a cross-sectional observation. Thus, the results did not establish a causal relationship between them. Fourth, the subjects were administered a questionnaire, their previous history of influenza was not checked, and 17% of subjects were excluded from the analysis because of failure to provide paired sera or appropriate data. Fifth, the neutralizing antibody test evaluated antibody titers based on the presence of inhibition of virus multiplication in cells. It was difficult to confirm an infection or the time of infection by using only the neutralizing antibody test because isolation of the virus itself was necessary to verify an infection with influenza viruses.

In conclusion, this study suggested the possibility that the first avian influenza H5N2 infection was transmitted to humans through exposure to infected chickens, and that a history of seasonal influenza vaccination and an age of over 40 years might be associated with positivity for H5N2-neutralizing antibody. Considering the serious impact of an influenza pandemic on public health, further epidemiological and virological examinations are recommended for the benefit of the global community.
